# Validating the biological origin of *in vivo* fractional intracellular volume from VERDICT-MRI in the prostate

**DOI:** 10.1186/s41747-026-00797-w

**Published:** 2026-09-09

**Authors:** Marta Masramon, Manju Mathew, Saurabh Singh, Thomy Mertzanidou, Joey Clemente, Adam Retter, Natasha Thorley, Adam Phipps, Thomas Parry, Marianti-Vasiliki Papoutsaki, Lorna Smith, Francesco Grussu, Veeru Kasivisvanathan, Alistair Grey, Eoin Dinneen, Greg Shaw, Dominic Patel, Lucy Caselton, Caroline M. Moore, David Atkinson, Aiman Haider, Alex Freeman, Daniel C. Alexander, Shonit Punwani, Eleftheria Panagiotaki

**Affiliations:** 1 Hawkes Institute, UCL, London, UK; 2 Centre for Medical Imaging, UCL, London, UK; 3 Department of Radiology, UCLH, London, UK; 4 Institute of Cardiovascular Sciences, UCL, London, UK; 5 Vall d’Hebron Institute of Oncology, Barcelona, Spain; 6 Division of Surgery and Interventional Sciences, UCL, London, UK; 7 Department of Urology, UCLH, London, UK; 8 Department of Urology, Barts Health NHS Trust, London, UK; 9 Research Department of Oncology, UCL, London, UK; 10 ARC Operation and Strategic Development Group, UCL, London, UK; 11 Department of Pathology, UCLH, London, UK

**Keywords:** Biomarkers, Diffusion magnetic resonance imaging, Multiparametric magnetic resonance imaging, Prostatic neoplasms, Prostatectomy

## Abstract

**Objective:**

Noninvasive microstructure estimates using magnetic resonance imaging (MRI) show potential in improving prostate cancer (PCa) diagnosis; however, histological validation is necessary.

**Materials and methods:**

We used data from the Histo-MRI trial (NCT04792138). Participants with biopsy-confirmed PCa underwent multiparametric (mp) MRI and “vascular, extracellular, and restricted diffusion for cytometry in tumors” (VERDICT)-MRI before radical prostatectomy. Personalized molds enabled matching prostatectomy histology with corresponding VERDICT-MRI images. Comparisons between histological epithelial cell density measures and MRI were made on a region-of-interest (ROI) basis using Pearson correlation coefficient (*r*) with 95% confidence interval (CI). Differences between benign tissue and clinically significant PCa were analyzed using the area under the receiver operating characteristic curve (AUC).

**Results:**

Fifty-two participants, aged 65 ± 6 years (mean ± standard deviation), with Gleason grade ≥ 3 + 4, were included. ROI analysis showed stronger correlations of VERDICT fractional intracellular volume (fIC) with epithelial fraction, epithelial cell density, and cell density (*r* = 0.784 [0.70, 0.84], 0.747 [0.66, 0.81], 0.711 [0.60, 0.79] than VERDICT ADC (*r* = -0.639 [-0.54, -0.71], -0.609 [-0.51, -0.69], -0.587 [-0.45, -0.69]), or mpMRI ADC (*r* = -0.357 [-0.18, -0.51], -0.315 [-0.15, -0.46], -0.266 [-0.08, -0.43]), respectively (*p* < 0.001). VERDICT fIC showed similar differences between benign and clinically significant PCa (0.16 ± 0.10 to 0.51 ± 0.13, AUC = 0.99, *p* < 0.001) to epithelial fraction (0.21 ± 0.08 to 0.43 ± 0.12, AUC = 0.94, *p* < 0.001).

**Conclusion:**

VERDICT fIC showed clinical potential as a biomarker of epithelial cell density.

**Key Points:**

- ***Question*** Can VERDICT-MRI fIC provide a biologically interpretable, noninvasive imaging biomarker of epithelial cell density in the prostate?
- ***Findings*** VERDICT fIC showed stronger correlations with histological epithelial fraction, cell density, and epithelial density than both VERDICT-derived ADC and mpMRI ADC.
- ***Relevance statement*** These findings support *in vivo* VERDICT fIC as a noninvasive biomarker of prostate epithelial microstructure and show promise for wider clinical use, improving the detection rate of clinically significant PCa, reducing unnecessary biopsies, and allowing better risk stratification.

**Graphical Abstract:**

**Figure:**
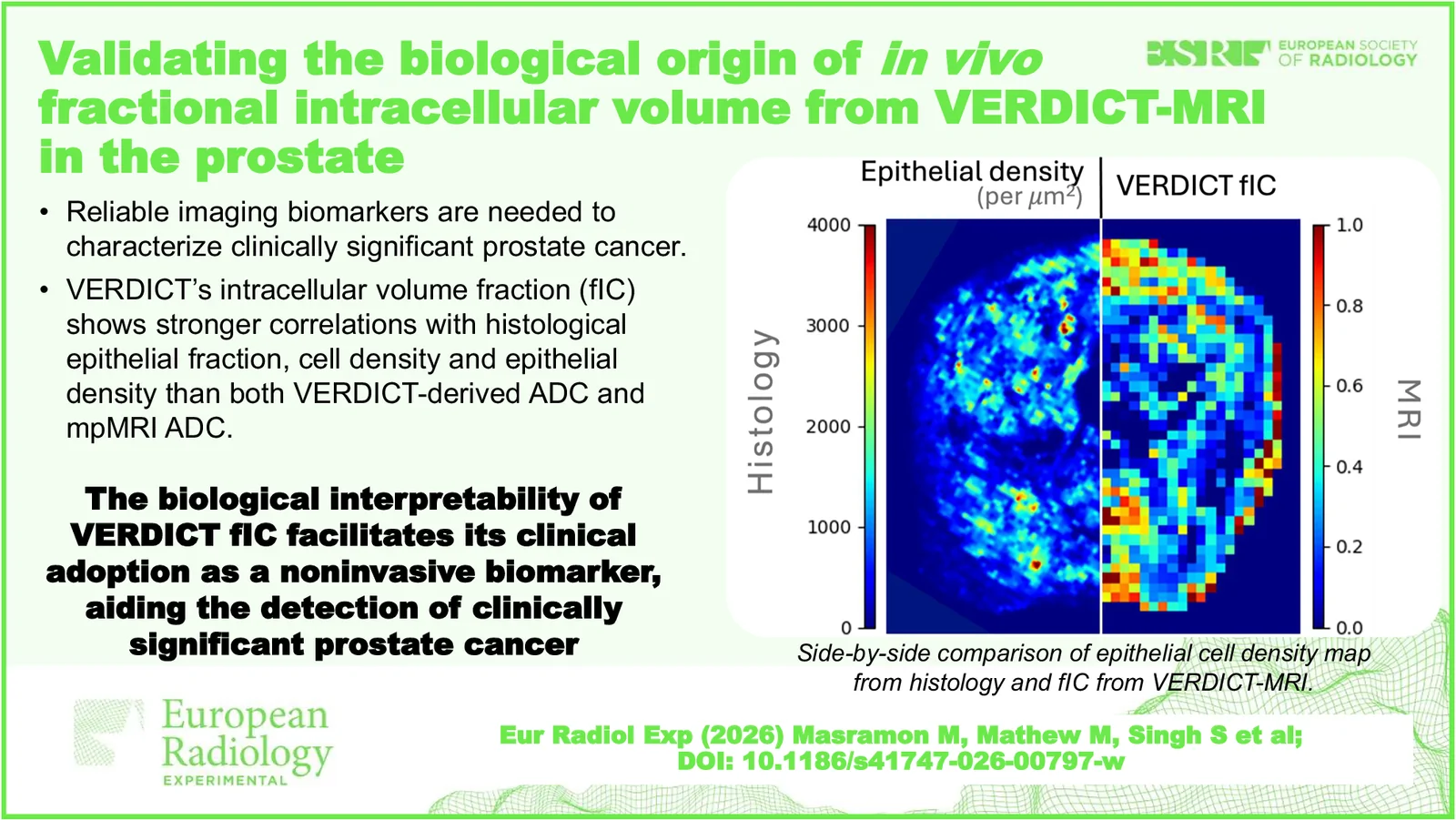

## Background

Multiparametric magnetic resonance imaging (mpMRI) plays a crucial role in the diagnosis and management of prostate cancer (PCa) by aiding in detection, localization, risk stratification, and staging of tumors. Despite its advantages, mpMRI has limited specificity [1] and insufficient sensitivity, missing 10–20% of tumors [2, 3]. Improving detection of clinically significant lesions requires an understanding of how MRI signals relate to histology and ensuring that these signals are specific to relevant histological features such as epithelial cell density, an increase of which is a key descriptor of adenocarcinoma—the most common type of PCa [4]. The apparent diffusion coefficient (ADC), a metric derived from the diffusion-weighted MRI acquisition within mpMRI, is routinely used to examine tissue microstructure. However, ADC compounds all physiological changes affecting water diffusion into a single measure, leading to poor discrimination of clinically significant PCa (csPCA) from clinically insignificant/benign change [5, 6].

To address this limitation, specialized diffusion-weighted MRI protocols combined with computational modeling offer a more precise characterization of tissue microstructure by examining specific features such as cell size and density [7]. The Vascular, Extracellular, and Restricted Diffusion for Cytometry in Tumors (VERDICT) framework is based on a biophysical model of diffusion in tumors [8]. It considers three water compartments—intracellular, vascular, and extracellular-extravascular—and estimates the volume fraction of each along with their diffusion coefficients. The fractional intracellular volume (fIC) derived from VERDICT has been explored in several studies (NCT02689271 [9–20], NCT04792138 [21], NCT05017181) and has shown promise as an imaging biomarker when used with mpMRI, achieving higher accuracy in detecting csPCa (area under the receiver operating characteristic curve (AUC) = 0.96) than ADC maps (AUC = 0.85) [10]. It has also demonstrated statistically significant differences between true-positive and false-positive lesions [13], with potential to reduce negative biopsies.

This study aims to verify the biological interpretation of *in vivo* VERDICT fIC. We use personalized three-dimensional-printed prostate molds to match whole-mount prostatectomy slices with *in vivo* VERDICT-MRI. We aim to validate the assumption of the VERDICT model that fIC correlates with epithelial cell density. We test our hypothesis by performing region of interest (ROI)-based comparisons of MRI and histology-derived measures of cell density and epithelial fraction. We also analyze differences between tissue composition and MR parameters in benign and csPCa tissue.

## Materials and methods

### Study participants

**Fig. 1:**
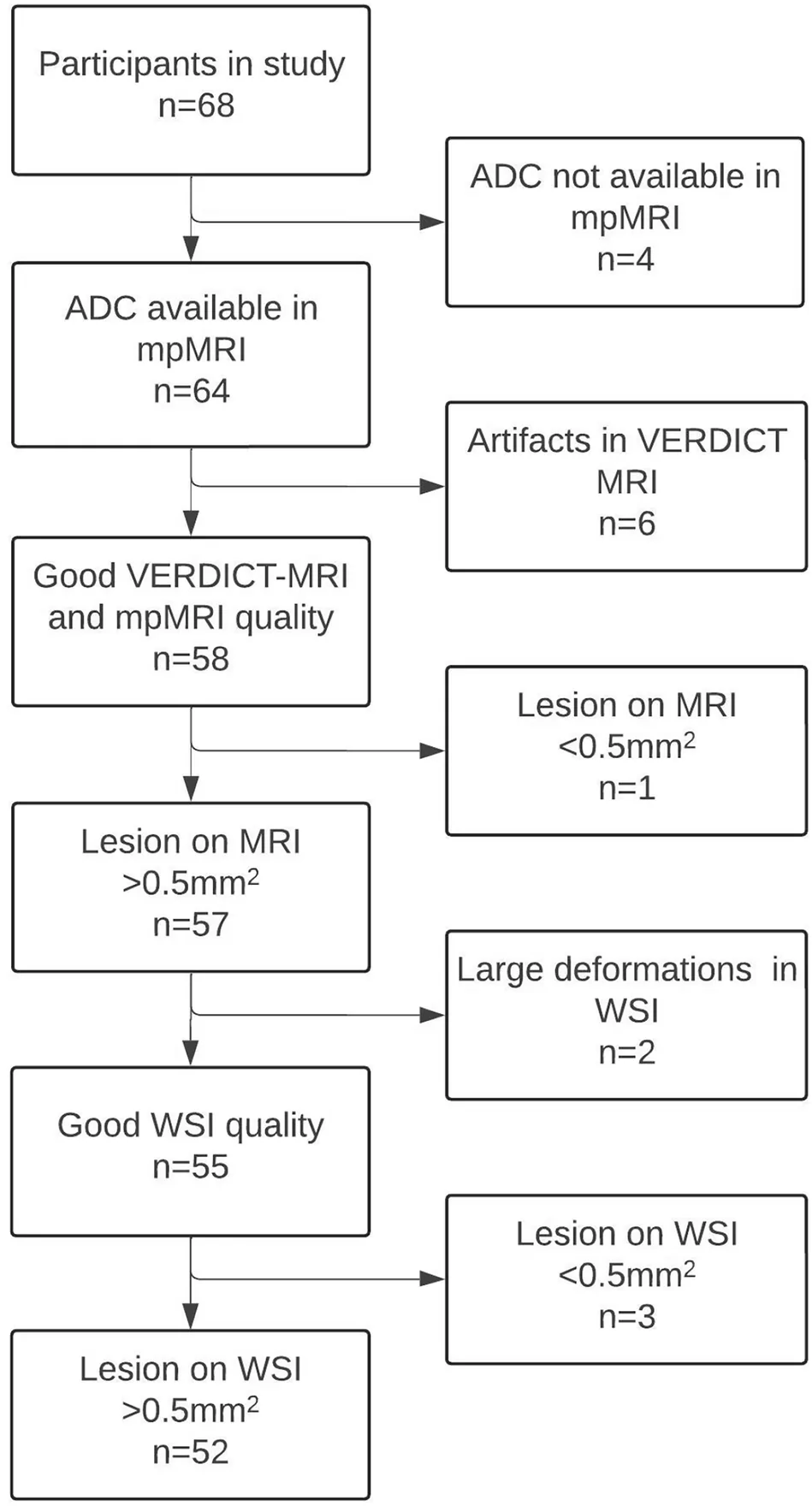
Participant selection flowchart. ADC, Apparent diffusion coefficient; VERDICT, Vascular, extracellular, and restricted diffusion for cytometry in tumors; WSI, Whole-slide imaging

Ethical approval for this prospective study was granted by the London-Queen Square Research Ethics Committee (19/LO/1803) on 23 January 2020. This study was performed in accordance with the Declaration of Helsinki. Patients were involved in research and ethics committee meetings reviewing study design and protocol before recruitment. We analyzed data from the Histo-MRI clinical trial (registered with ClinicalTrials.gov, NCT04792138, first posted 10/03/2021 and ongoing since 2020 with consecutive sampling; amendments were made to increase sample size, 15/09/2023), where men with a clinical suspicion of PCa are scanned using VERDICT-MRI. Patients attending a One-stop PCa clinic at University College London Hospital were screened against the inclusion and exclusion criteria, and eligible patients were invited. All screened participants were provided with Participant Information Leaflets detailing the study's objectives and their involvement; those interested provided informed written consent. The inclusion criterion was men aged 18+ with no MRI contraindications. Exclusion criteria included inability to undergo MRI, inability to provide informed consent, or prior/ongoing PCa treatment (surgery, radiotherapy, hormone treatment). MRI files were anonymized using DicomCleaner [22], and whole-slide images were saved under study identifiers. Here, we analyze patients who underwent prostatectomy (*n* = 68). Participants missing ADC on the mpMRI, or with large MRI artifacts, histology deformations, or lesions smaller than the diffusion-weighted imaging voxel size (0.5 mm²), are excluded (Fig. 1).

### Tissue preparation

To ensure slice-to-slice correspondence between histology and MR images, custom molds were created from manual prostate contours on pre-operative T2W axial images using Horos [23]. Reference slices, set as the plane with the largest lesion detected on the mpMRI, were chosen and used to place cutting guides within the mold. After removal of the prostates during radical prostatectomy, specimens were inked to denote laterality and silicon catheters inserted through the urethra. Prostates were sealed in their molds and placed in neutral buffered formalin for 24-h fixation. The full specimen handling protocol is described by Bourne et al [24].

For histological analysis, a histopathologist (MMathew—5 years of experience) cut the reference slice following the cutting guides, spaced 5 mm apart. The remainder of the prostate was progressively sliced at 5 mm increments, matching MRI slice resolution. Tissue sections were processed per standard protocol, using hematoxylin and eosin dyes [25].

### MRI acquisition

The MRI acquisitions included mpMRI and VERDICT-MRI [21]. VERDICT scans were performed on a 3-T MRI scanner (Achieva or Ingenia; Philips, Best, the Netherlands). A pulse-gradient spin-echo sequence was acquired in the axial plane using a 32-channel cardiac coil (Achieva) or body coils (Ingenia); *b*-values included 90, 500, 1,500, 2,000, and 3,000 s/mm^2^ obtained in three orthogonal directions, with a *b* = 0 s/mm^2^ image for every *b*-value. This was used to normalize T2 and T1 dependencies. Further sequence parameters were as follows: phase-encoding direction anterior/posterior; field of view = 220 × 220 mm^2^; acquisition matrix = 168 × 168; and acquired voxel size = 1.31 × 1.31 × 5.0 mm^3^. Each *b*-value had a corresponding repetition and echo time. VERDICT scans lasted 11:35 min:s (Achieva) or 17:41 min:s (Ingenia). For more details, see Singh et al [21].

ADC maps were derived using voxel-wise mono-exponential fitting. We used diffusion-weighted images with *b*-values up to 1,000 s/mm^2^ (1.5 or 3 T, see Supplementary Tables S1 and S2) from the clinical mpMRI (mpMRI ADC). To derive the VERDICT ADC, we used *b*-values of 0, 90, 500, and 1,500 s/mm^2^ from the VERDICT scans, as in Rogers et al [15].

Radiologists (A.R.—‘in-training’, N.T.—5 years of experience, S.P.—20 years of experience) drew PCa ROIs on the MR images in consensus using MIM 7.2.9 [26], using the clinical mpMRI and biopsy reports to locate the main lesion. Benign regions were identified on histology and manually transferred onto MRI to avoid inclusion of cancer and benign confounding conditions such as hyperplasia, cystic atrophy and inflammation.

### VERDICT prostate model

The VERDICT mathematical model assumes no exchange and similar T2 values between water populations [8]. The normalized signal S is the sum of three parametric models:

$$S=\,{\sum}_{{i=f}_{{IC}},{f}_{{VASC}}{,f}_{{EES}}}{f}_{i}{S}_{i}$$

where ${f}_{i}$ is the volume fraction and ${S}_{i}$ the proportion of signal with no diffusion weighting of each compartment. For prostate tissue, the intracellular compartment is modeled as restricted diffusion in impermeable spheres, the vascular compartment as isotropically restricted water in cylinders with uniformly distributed orientations and zero diameter, and the extracellular extravascular compartment as an isotropic diffusion tensor model. We use fixed diffusion values ${d}_{IC} = {d}_{EES} = 2 \cdot 10 ^{-9} mm^2/s$ and ${d}_{{{\mathrm{VASC}}}}=8\cdot {10}^{-9}{{{\mathrm{mm}}}}^{2}/{{{\rm{s}}}}$ as in previous work [10, 21] and fit the model using the VERDICT-AMICO algorithm [9].

### Histological analysis

A semiautomated pipeline was created to process the histology images and produce quantitative maps. The appearance of the Hematoxylin and Eosin staining on histology is often highly variable [27]. To normalize the images, the brightness, contrast, and saturation of stained whole-slide histopathology was standardized. Shrinkage was accounted for using a correction factor of 1.15, proposed by Schned et al and confirmed by Jonmarker et al for specimens processed with formalin fixation [28, 29].

**Fig. 2:**
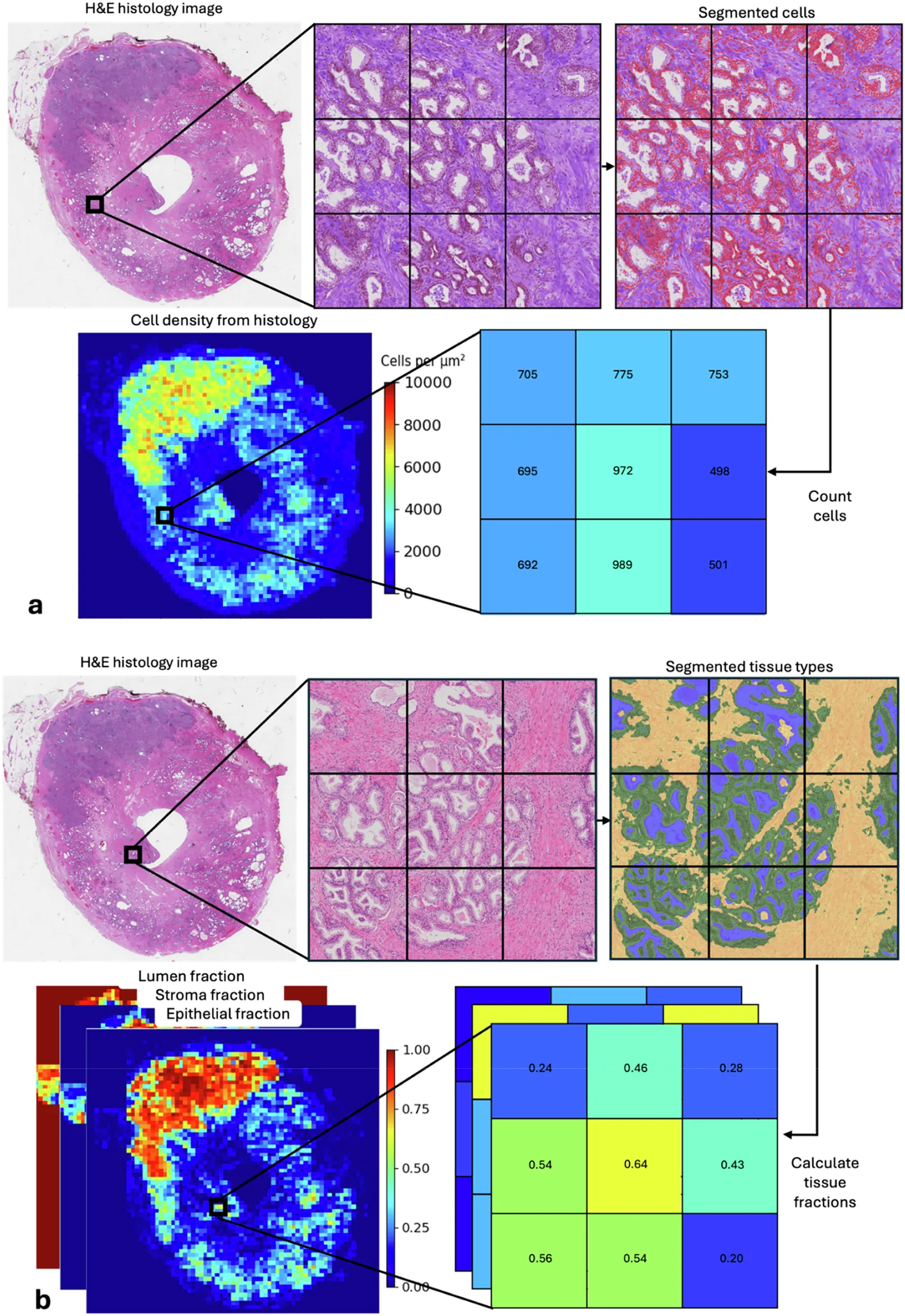
Pipelines to obtain cell density and tissue fraction maps from histology. High-resolution images are divided into 512-pixel-wide patches; each patch value represents a pixel in the resulting map. **a** Nuclei are segmented (outlined in red), counted, and normalized by the total area of the patch. **b** Tissue is segmented into epithelium (green), lumen (blue), and stroma (yellow), then the proportion of each tissue type is calculated per patch

**Fig. 3:**
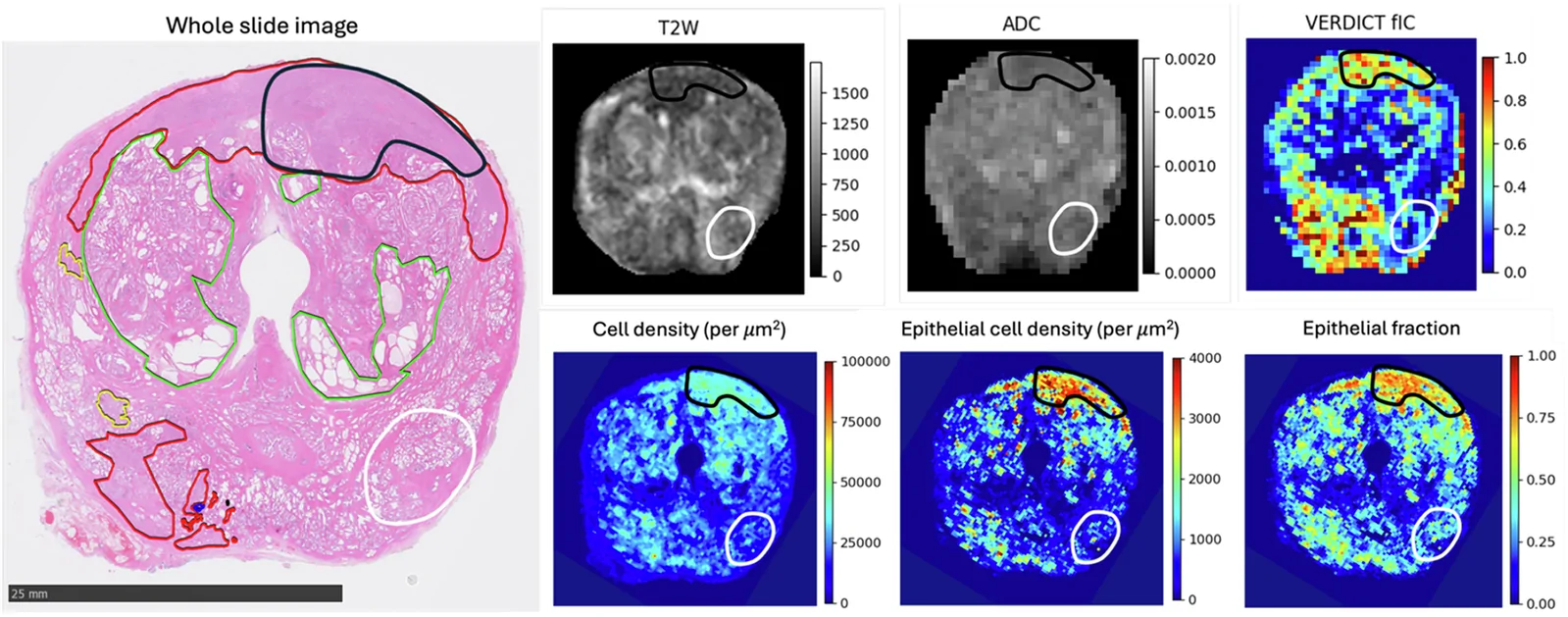
Representative example: a 67-year-old man with prostate-specific antigen levels of 10.89 ng/mL at referral, prostate volume of 36 mL and Likert score 3. WSI (left) and corresponding masked MRI and histologically derived maps (T2-weighted imaging and ADC from mpMRI and fIC from VERDICT-MRI; cell density, epithelial cell density and epithelial fraction from histology). Relevant tissue morphologies are contoured on the WSI: benign prostatic hyperplasia (green), inflammation (blue), Gleason 3 + 3 (yellow) and Gleason 3 + 4 (red). All images show the ROIs used for quantitative analysis: benign (white) and Gleason 3 + 4 (black). ADC, Apparent diffusion coefficient; fIC, Fractional intracellular volume; ROI, Region of interest; VERDICT, Vascular, Extracellular, and Restricted Diffusion for Cytometry in Tumors; WSI, Whole-slide imaging

**Fig. 4:**
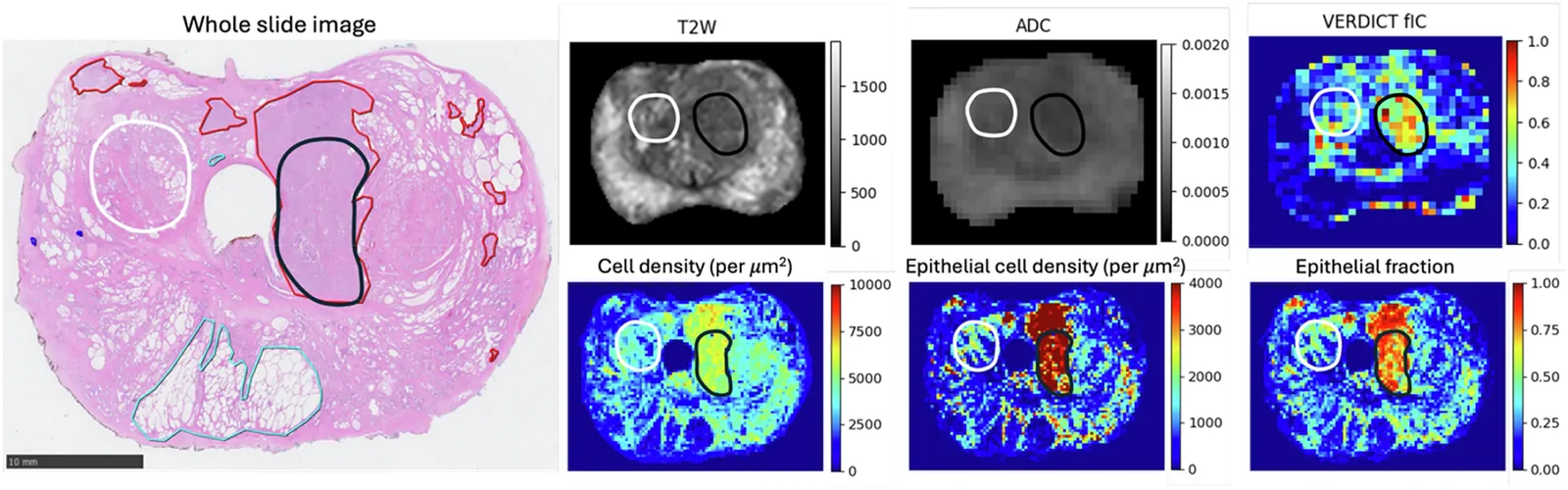
Representative example: a 62-year-old man with prostate-specific antigen levels of 7.75 ng/mL at referral, prostate volume of 37 mL and Likert score 3. WSI (left) and corresponding masked MRI and histologically derived maps (T2-weighted imaging and ADC from mpMRI and fIC from VERDICT-MRI; cell density, epithelial cell density and epithelial fraction from histology). Relevant tissue morphologies are contoured on the WSI: cystic atrophy (cyan), inflammation (blue) and Gleason 3 + 4 (red). All images show the ROIs used for quantitative analysis: benign (white) and Gleason 3 + 4 (black). ADC, Apparent diffusion coefficient; fIC, Fractional intracellular volume; ROI, Region of interest; VERDICT, Vascular, extracellular, and restricted diffusion for cytometry in tumors; WSI, Whole-slide imaging

The following steps were done on a patch-by-patch basis (535 × 535 mm) on QuPath [30]. Cell density was calculated from cell nuclei segmentations (Fig. 2a). Tissue fractions were obtained by segmenting each patch into stroma, lumen, and epithelial tissue using a pixel classifier trained on expert annotations (histopathologist M.M.—5 years of experience; Fig. 2b). Epithelial cell density was calculated by weighing cell density by epithelial fraction. All steps of this pipeline were visually checked by a single histopathologist (M.M.). Lesions in histology were contoured and graded by histopathologists (primary annotator M.M. and senior reviewers A.F.—20 years of experience—and A.H.—9 years of experience). Figures 3 and 4 show representative examples.

### Statistical analysis

The primary outcome of this study was the correlation (with 95% confidence intervals) of features from histology and MR-derived maps. In each sample, ROIs were drawn in cancerous and benign areas of tissue using consistent anatomical landmarks across MR and histology. Cancerous ROIs were based on radiologist annotations on mpMRI; benign ROIs were drawn directly on histology (to exclude any confounding pathology) and transferred to MRI. The average value of each ROI was used. Statistical significance was based on 95% confidence intervals. Although our study was not powered to test for differences between correlations, an exploratory comparison is provided in Supplementary Table S3. All analyses were performed in Python. The secondary outcome was the AUC between csPCa and benign tissue.

## Results

### Participant characteristics

From the total of 68 participants, four were excluded due to lack of ADC maps on mpMRI, six due to image artifacts in MRI, two due to deformations in histology, three due to small lesions on histology, and one because of small lesions on MRI (< 0.5 mm^2^, Fig. 1). Participant and lesion characteristics of the remaining participants (*n* = 52) are summarized in Table 1. The age of these men was 65 years ± 6 (mean ± standard deviation). The mean prostate-specific antigen level was 8.4 ng/mL ± 6.1. The mean time between prostate-specific antigen test and prostatectomy was 56 ± 39 days, and the mean time between mpMRI and VERDICT-MRI was 108 ± 115 days. The mean time between VERDICT-MRI and prostatectomy was 48 ± 44 days. A single PCa and a benign ROI were taken from each patient. Lesions have Gleason grades 3 + 4 (*n* = 37), 3 + 4 + 5 (*n* = 4), 4 + 3 (*n* = 7), 4 + 3 + 5 (*n* = 2), 4 + 4 (*n* = 1), and 4 + 5 (*n* = 1).

**Table 1:** Summary of the participants’ and the lesions’ characteristics

Participant/lesion characteristics	Mean ± standard deviation (range)
Age (years)	65 ± 6 (47–76)
PSA level (ng/mL)	8.4 ± 6.1 (2.4–36.0)
Time between PSA and surgery (days)	56 ± 39 (0–159)
Time between mpMRI and VERDICT-MRI (days)	108 ± 115 (0–494)
Time between VERDICT-MRI and surgery (days)	48 ± 44 (1–159)
	**Count (percentage)**
Ethnicity	
White British	25 (48.0%)
Other White	7 (13.5%)
Asian Indian	2 (3.8%)
Other Asian	2 (3.8%)
Black African	5 (9.6%)
Black Caribbean	1 (1.9%)
Other Black	1 (1.9%)
Other ethnic group	2 (3.8%)
Not stated/Not yet asked	7 (13.5%)
Gleason grade of main lesion (primary + secondary + tertiary pattern)	
3 + 4	37 (71.2%)
3 + 4 + 5	4 (7.7%)
4 + 3	7 (13.5%)
4 + 3 + 5	2 (3.8%)
4 + 4	1 (1.9%)
4 + 5	1 (1.9%)

Ethnicity was acquired from hospital records; participants self-reported from a list of options

*mpMRI* Multiparametric magnetic resonance imaging, *PSA* Prostate-specific antigen, *VERDICT* Vascular, extracellular, and restricted diffusion for cytometry in tumors

### Comparison of VERDICT fIC and mpMRI ADC against histological features in benign and csPCa ROIs

**Fig. 5:**
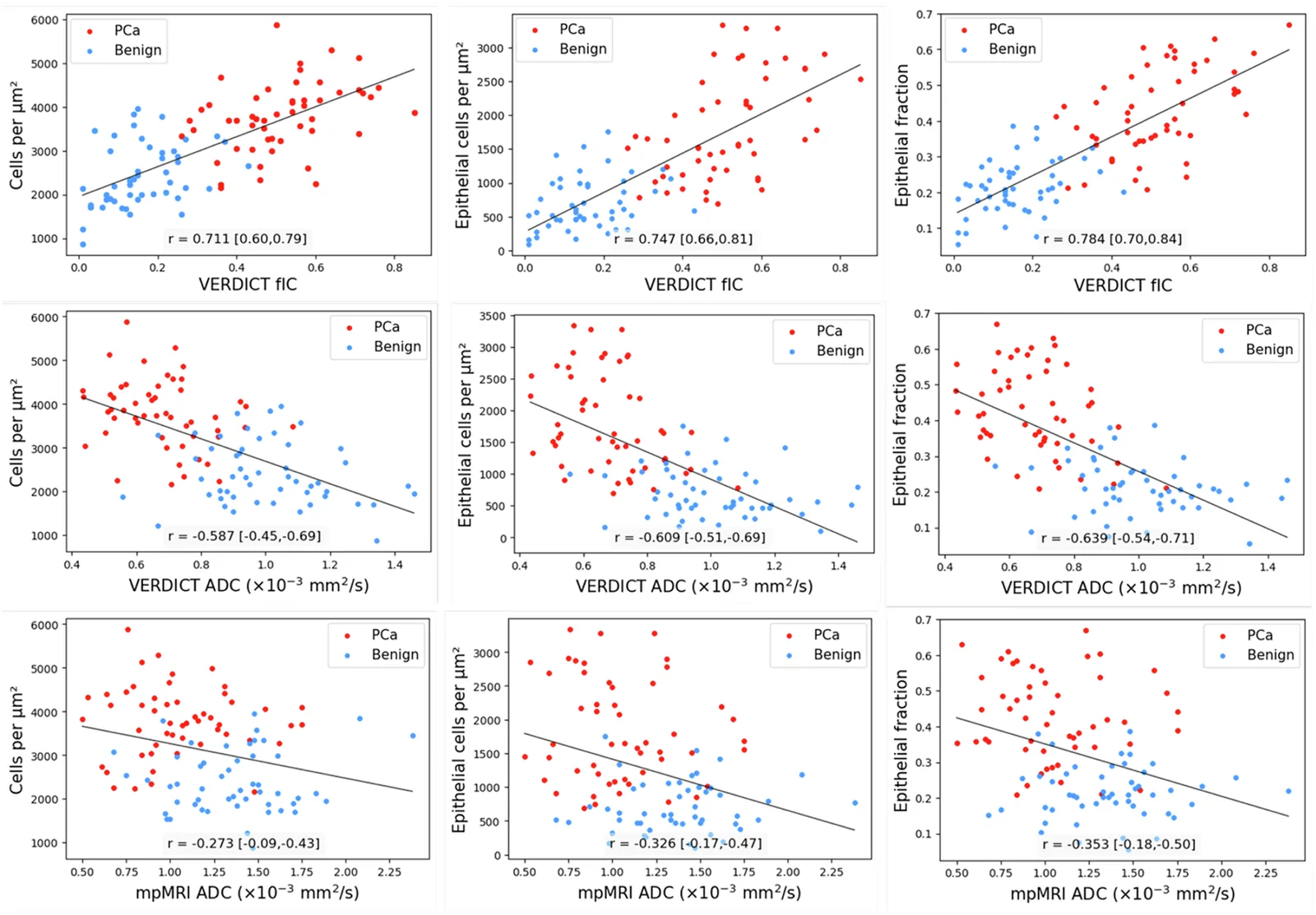
Linear correlation between MRI maps (VERDICT fIC and ADC, mpMRI ADC) and histological parameters (cell density, epithelial cell density and epithelial fraction). ADC, Apparent diffusion coefficient; fIC, Fractional intracellular volume; mpMRI, Multiparametric MRI; *r* Pearson correlation coefficient with 95% confidence interval from bootstrapping in brackets; VERDICT, Vascular, extracellular, and restricted diffusion for cytometry in tumors

When analyzing benign and cancerous ROIs in the full cohort, fIC correlates most strongly with the histological epithelial fraction (*r* = 0.784 [0.70, 0.84], *p* < 0.001). It correlates with lower strength to epithelial cell density (*r* = 0.747 [0.66, 0.81], *p* < 0.001) and cell density (*r* = 0.711 [0.60, 0.79], *p* < 0.001). VERDICT ADC maps show weaker correlations to these histological parameter maps (*r* = -0.639 [-0.54, -0.71], *p* < 0.001, -0.609 [-0.51, -0.69], *p* < 0.001 and -0.587 [-0.45, -0.69], *p* < 0.001, respectively) while mpMRI ADC exhibits the weakest correlations (*r* = -0.357 [-0.18, -0.51], *p* < 0.001, -0.315 [-0.15, -0.46], *p* < 0.001 and -0.266 [-0.08, -0.43], *p* = 0.005, respectively). In all cases, the correlation with epithelial fraction is strongest. These findings are summarized in Table 2 and illustrated in Fig. 5.

**Table 2:** Correlation between MR maps and histological parameters

MRI map	Pearson *r* value		
	Cell density	Epithelial cell density	Epithelial fraction
VERDICT fIC	0.711 [0.60, 0.79], *p* < 0.001	0.747 [0.66, 0.81], *p* < 0.001	0.784 [0.70, 0.84], *p* < 0.001
VERDICT ADC	-0.587 [-0.45, -0.69], *p* < 0.001	-0.609 [-0.51, -0.69], *p* < 0.001	-0.639 [-0.54, -0.71], *p* < 0.001
mpMRI ADC	-0.266 [-0.08, -0.43], *p* = 0.005	-0.315 [-0.15, -0.46], *p* < 0.001	-0.357 [-0.18, -0.51], *p* < 0.001

Data in brackets are 95% confidence intervals from bootstrapping. The *p*-values reflect individual correlation fits

*ADC* Apparent diffusion coefficient, *fIC* Intracellular volume fraction, *mpMRI* Multiparametric magnetic resonance imaging, *VERDICT* Vascular, extracellular, and restricted diffusion for cytometry in tumors

### Comparison of MRI-derived parameters and tissue composition in benign and cancerous prostate regions

Table 3 presents how fIC, ADC, and tissue composition differ between benign and csPCa prostate regions. Tissue composition changes between benign and csPCa are characterized by an increase in epithelial fraction (0.21 ± 0.08 to 0.43 ± 0.12, AUC = 0.94 [0.89, 0.98], *p* < 0.001) and a decrease in both lumen fraction (0.24 ± 0.13 to 0.12 ± 0.04, AUC = 0.86 [0.79, 0.93], *p* < 0.001) and stroma fraction (0.57 ± 0.16 to 0.49 ± 0.13, AUC = 0.68 [0.57, 0.78], *p* < 0.001). Cell density and epithelial cell density are both higher in csPCa (3780 ± 790 and 1810 ± 750 cells per ${{{\mathrm{\mu m}}}}^{2}$, respectively) compared to benign tissue (2,360 ± 780 and 680 ± 380 cells per ${\mu m}^{2}$, respectively).

**Table 3:** Comparison of MRI parameters and tissue composition in benign and csPCa regions

Parameter map	Benign	csPCa	AUC	*p*-value
Cell density (per ${\mu m}^{2}$)	2,360 ± 780	3,780 ± 790	0.90 [0.84, 0.95]	< 0.001
Epithelial cell density (per ${{{\mathrm{\mu m}}}}^{2}$)	680 ± 380	1810 ± 750	0.92 [0.87, 0.97]	< 0.001
Epithelial fraction	0.21 ± 0.08	0.43 ± 0.12	0.94 [0.89, 0.98]	< 0.001
Stroma fraction	0.57 ± 0.16	0.49 ± 0.13	0.68 [0.57, 0.78]	< 0.001
Lumen fraction	0.24 ± 0.13	0.12 ± 0.04	0.86 [0.79, 0.93]	< 0.001
VERDICT fIC	0.16 ± 0.10	0.51 ± 0.13	0.99 [0.97, 1.00]	< 0.001
VERDICT ADC (${\times 10}^{-3}{{{{\rm{mm}}}}}^{2}/{{{\rm{s}}}}$)	1.00 ± 0.19	0.68 ± 0.14	0.92 [0.87, 0.97]	< 0.001
mpMRI ADC (${\times 10}^{-3}{{{{\rm{mm}}}}}^{2}/{{{\rm{s}}}}$)	1.36 ± 0.32	1.05 ± 0.31	0.76 [0.66, 0.85]	< 0.001

Data in the benign and csPCa columns are mean values ± standard deviation. AUC values for differentiating benign from csPCa tissue are shown. Data in brackets are 95% confidence intervals from bootstrapping. A *p*-value < 0.05 represents a significant difference from the null hypothesis that AUC is 0.5

*ADC* Apparent diffusion coefficient, *csPCa* Clinically significant prostate cancer, *fIC* Intracellular volume fraction, *mpMRI* Multiparametric magnetic resonance imaging, *VERDICT* Vascular, extracellular, and restricted diffusion for cytometry in tumors

As in the case of cell density, epithelial cell density and epithelial fraction, VERDICT fIC shows a statistically significant difference between benign and csPCa tissue (0.16 ± 0.10 to 0.51 ± 0.13, AUC = 0.99 [0.97, 1.00], *p* < 0.001). Mean VERDICT ADC and mpMRI ADC values in benign and csPCa regions are 1.00 ± 0.19 ${\times 10}^{-3}$ to 0.68 ± 0.14 ${\times 10}^{-3}{{{{\rm{mm}}}}}^{2}/{{{\rm{s}}}}$ and 1.36 ± 0.32 ${\times 10}^{-3}$ to 1.05 ± 0.31 ${\times 10}^{-3}{{{{\rm{mm}}}}}^{2}/{{{\rm{s}}}}$, respectively.

## Discussion

This study investigated the biological origin of the fIC from VERDICT-MRI using three-dimensional personalized molds to enable accurate histological comparison. ROI analysis showed a strong correlation of fIC with epithelial fraction (*r* = 0.784) and histological cell density (*r* = 0.711). This supports our hypothesis that the fIC estimate is positively correlated with epithelial cell density.

The VERDICT model assumes that the intracellular component captures the signal solely from epithelial cells, while the signal from stromal cells and lumen is accounted for by the extracellular extravascular component. Importantly, results show that fIC correlates better with epithelial fraction than cell density, which is a less effective Gleason Pattern predictor than tissue fractions [31]. Thus, our results provide converging evidence that VERDICT fIC reflects epithelial tissue composition (epithelial fraction) rather than generic cellularity (cell density) or diffusion restriction. This aligns with glandular changes characteristic of prostate adenocarcinoma, namely the proliferation of epithelial cells [4]. From a clinical perspective, fIC may provide complementary information to mpMRI, particularly in indeterminate lesions (*e.g*., PI-RADS 3). This could reduce unnecessary biopsies and improve risk stratification by providing a quantitative, biologically interpretable imaging biomarker.

Previous ROI-based studies that describe the relationship between histological cell density and ADC in PCa report correlations between *r* = -0.50 and *r* = -0.695 [32–35]. Our findings for ADC from mpMRI show a correlation of r = -0.266. We speculate that the variability in these results is due to differences in the b-values, scanner manufacturers, and diffusion sequences used to calculate ADC maps, as well as cell counting algorithms. Notably, the higher correlation of ADC with epithelial fraction presented here (*r* = -0.357) has been previously noted by Chatterjee et al who reported correlations with cell count and epithelial fraction in fixed tissue of -0.598 and -0.647, respectively [31]. They also showed that both correlations are stronger in fresh tissue.

Our results also show that VERDICT ADC is more strongly correlated to histological measures of cell density and epithelial fraction than mpMRI ADC (-0.587 and -0.639 *versus* -0.273 and -0.353). We believe that this is primarily due to using a consistent imaging sequence. In this study, we derive the VERDICT ADC from diffusion-weighted images with b-values 0, 90, 500, and 1,500 s/mm2, each with a given TR and TE, while each mpMRI ADC uses a different number of diffusion-weighted images, with different b-values (see Supplementary Table S1) and inconsistent TR and TE. The fact that VERDICT fIC outperforms VERDICT ADC highlights the importance of complex microstructural models to discriminate the relevant signal components. ADC is a bulk diffusion metric influenced by all cell environments, not specific to tissue changes that occur in PCa, and so lacks precise biophysical interpretation.

Further analysis investigated differences in tissue composition and MR-derived parameters between benign and csPCa ROIs. Consistent with previous findings [31], csPCa regions exhibit a higher epithelial fraction and lower luminal fraction compared to benign tissue (0.21 ± 0.08 and 0.24 ± 0.13 *versus* 0.43 ± 0.12 and 0.12 ± 0.04). VERDICT fIC demonstrates similar changes to the epithelium, with values increasing from 0.16 ± 0.10 in benign regions to 0.51 ± 0.13 in csPCa. The highest AUC values correspond to epithelial fraction and fIC (0.94 [0.89, 0.98] and 0.99 [0.97, 1.00], respectively), highlighting their ability to differentiate between tissue types, while both VERDICT ADC and mpMRI ADC exhibit lower discriminatory performance (AUC = 0.92 [0.87, 0.97] and 0.76 [0.66, 0.85], respectively). The parallel increase in epithelial fraction and fIC across benign and csPCa regions provides direct biological validation that fIC tracks dominant histological transformations in PCa, the replacement of luminal space by epithelial tissue [4].

We note certain limitations in our study. Resection of the prostate creates shape changes due to removal of mechanical tension and compression and hemodynamic pressure [36], dehydration and embedding of the tissue during processing cause tissue shrinkage; and thin sectioning may cause further deformations [24]. This compromises the histology-MR mapping and affects the distribution of tissue fractions; future work could incorporate deformable registration or three-dimensional histology reconstruction to mitigate these effects. Lesions smaller than 0.5 mm² were excluded from our analysis, as they fall below the resolution threshold of diffusion-weighted MRI; investigating whether VERDICT can detect such small lesions will require higher-resolution acquisitions or ultra-high-field imaging [37]. MpMRI was performed on various scanners while VERDICT-MRI was consistently obtained from Philips machines; assessing reproducibility across vendors and field strengths in future multi-center studies will be essential for clinical translation. The validation design of this study restricted our analysis to prostatectomy cases, likely inflating the observed AUCs between benign and cancerous regions and restricting generalizability to other cohorts, with the limited cohort size representing an additional constraint. Finally, while studies on active surveillance report median ADC reductions of -9.5% over 30–51 months [38] and -6.8% over 23 (IQR 14–24) months [39] in progressing tumors, the shorter interval in this study (mean ~3.5 months) is unlikely to substantially affect ADC or fIC comparisons, although minor effects of temporal variation cannot be excluded.

In conclusion, this study provides direct histological validation of the biological origin of the VERDICT fIC, supporting that it reflects epithelial tissue composition in PCa. By linking MRI measurements to underlying microstructure, our results enhance the interpretability of fIC and support its use as a specific, non-invasive biomarker of clinically significant disease. This improved specificity addresses a key limitation of conventional mpMRI and may facilitate more accurate detection and characterization of PCa in clinical practice. Future work will further investigate the relationship between the model’s parameters and histological metrics across different Gleason scores and growth patterns, with a focus on mpMRI-invisible lesions, in larger, multi-center cohorts using a range of scanner manufacturers.

## Supplementary information

**Additional File 1: Table S1.** Distribution of b-values used for calculation of ADC maps in clinical mpMRI. **Table S2.** Distribution of field strengths used for clinical mpMRI. **Table S3**. Comparison of correlations between MRI-derived metrics and histological tissue measures using Williams-modified Hotelling *t*-tests. **Fig. S1**. Representative example: a 67-year-old man with prostate-specific antigen levels of 10.89 ng/mL at referral, prostate volume of 36 mL and Likert Score 3. WSI (left) and corresponding masked MRI and histologically derived maps (fIC, fEES, fVASC and R from VERDICT-MRI; cell density, epithelial cell density and epithelial fraction from histology). fEES, Fractional extracellular extravascular space; fIC, Fractional intracellular volume; fVASC, Fractional vascular volume; R, Radius; VERDICT Vascular, extracellular, and restricted diffusion for cytometry in tumors; WSI, Whole-slide imaging. **Fig. S1**. Representative example: a 67-year-old man with prostate-specific antigen levels of 10.89 ng/mL at referral, prostate volume of 36 mL, and Likert Score 3. WSI (left) and corresponding masked MRI and histologically derived maps (fIC, fEES, fVASC and R from VERDICT-MRI; cell density, epithelial cell density and epithelial fraction from histology). fEES, Fractional extracellular extravascular space; fIC, Fractional intracellular volume; fVASC, Fractional vascular volume; R, Radius; VERDICT, Vascular, extracellular, and restricted diffusion for cytometry in tumors; WSI, Whole-slide imaging.

## Data Availability

The datasets analyzed within this study are not publicly available due to data protection laws but may be available from the corresponding author on reasonable request and subject to approval by the relevant institutional data protection office. The code used in this study is publicly available at https://github.com/Martamasramon/VERDICT-fIC-validation (commit 8e84b1eba228bc33c05143874db74ae9249da987).

## Abbreviations

ADC: Apparent diffusion coefficient
AUC: Area under the receiver operating characteristic curve
csPCa: Clinically significant prostate cancer
fIC: Fractional intracellular volume
mpMRI: Multiparametric MRI
MRI: Magnetic resonance imaging
PCa: Prostate cancer
ROI: Region of interest
VERDICT: Vascular, extracellular, and restricted diffusion for cytometry in tumors
