# Supplementary material for: Validating the biological origin of in vivo fractional intracellular volume from VERDICT-MRI in the prostate
Source: Eur Radiol Exp. 2026 Sep 9;10:123. doi: 10.1186/s41747-026-00797-w (PMC13558491; doi:10.1186/s41747-026-00797-w)
Supplement: Supplementary file 1 [file 41747_2026_797_MOESM1_ESM.pdf]

Validating the biological origin of *in vivo* fractional intracellular volume from VERDICT-MRI in the prostate

ELECTRONIC SUPPLEMENTARY MATERIAL

Supplementary material

A. Sequence parameter distribution of mpMRI

Tables S1 and S2 illustrate the heterogeneity of scanner and sequence parameters used to obtain ADC maps in the clinical mpMRI used in this study.

Table S1 Distribution of *b*-values used for calculation of ADC maps in clinical mpMRI

b-values (s/mm^2)	Number of patients
0, 1,000	6
0, 50, 800	3
0, 150, 500, 1,000	13
0, 150, 800, 1,000	5
0, 500, 800	1
50, 1,000	2
50, 150, 500, 1,000	19
50, 400, 800	1
50, 500, 1,000	1
100, 500, 1,000	1

Table S2 Distribution of field strengths used for clinical mpMRI

**Field strength (T)**	**Number of patients**
1.5	25
3	27

B. Statistical comparisons between correlations

Pearson correlation coefficients were computed between each MRI-derived metric (VERDICT fIC, VERDICT ADC, and mpMRI ADC) and histological measures of tissue composition (cell density, epithelial fraction, and epithelial density). Williams-modified Hotelling t-tests were used to compare dependent correlations sharing a common outcome variable, testing whether one MRI metric correlated significantly more strongly with each tissue measure than another.

Table S3 Comparison of correlations between MRI-derived metrics and histological tissue measures using Williams-modified Hotelling *t*-tests.

Tissue	Comparison	r_1	r_2	t	*p*-value
Density	mpMRI ADC vs VERDICT ADC	-0.274	-0.566	3.41	0.001
	VERDICT fIC vs VERDICT ADC	0.701	-0.566	9.46	<0.001
	VERDICT fIC vs mpMRI ADC	0.701	-0.274	7.18	<0.001
Epithelial	mpMRI ADC vs VERDICT ADC	-0.354	-0.627	3.37	0.001
	VERDICT fIC vs VERDICT ADC	0.775	-0.627	11.73	<0.001
	VERDICT fIC vs mpMRI ADC	0.775	-0.354	9.14	<0.001
Epithelial density	mpMRI ADC vs VERDICT ADC	-0.329	-0.604	3.33	0.001
	VERDICT fIC vs VERDICT ADC	0.745	-0.604	10.74	<0.001
	VERDICT fIC vs mpMRI ADC	0.745	-0.329	8.34	<0.001

r_1 and r_2 denote the Pearson correlation coefficients for the first and second predictor in each comparison, respectively; *t* denotes the test statistic; *p*-values are corrected for multiple comparisons using the Benjamini-Hochberg false discovery rate procedure. *ADC* Apparent diffusion coefficient. *fIC* Fractional intracellular volume, *mpMRI*: Multiparametric magnetic resonance imaging, *VERDICT* Vascular, Extracellular, and Restricted Diffusion for Cytometry in Tumors.

C. Additional VERDICT parameter maps

Figs. S1 and S2 extend manuscript Figs. 3 and 4, respectively, by visualizing VERDICT fFASC, fEES and R (radius) maps.

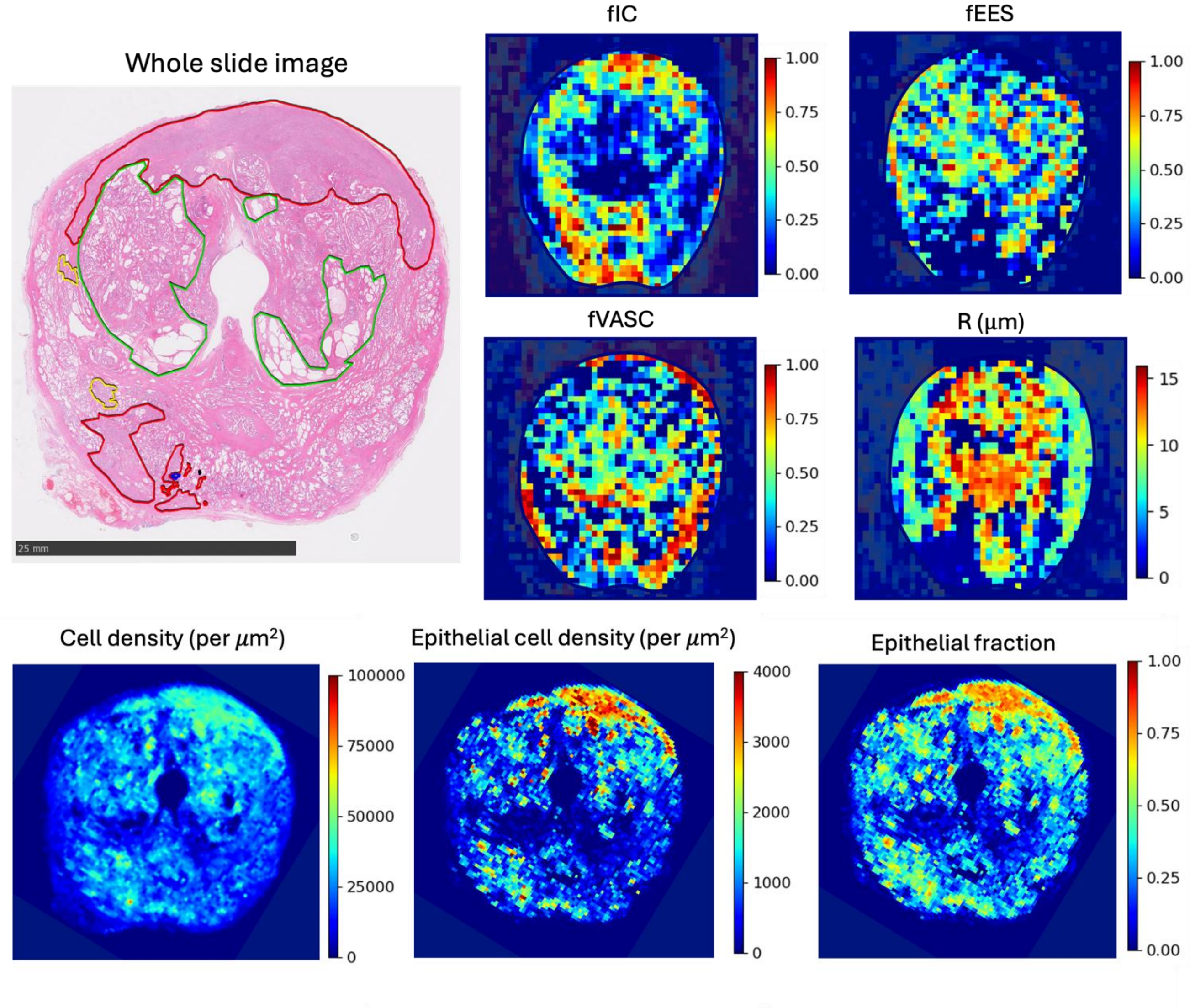

Fig. S1. Representative example: a 67-year-old man with prostate-specific antigen levels of 10.89 ng/mL at referral, prostate volume of 36 mL and Likert Score 3. WSI (left) and corresponding masked MRI and histologically derived maps (fIC, fEES, fVASC and R from VERDICT-MRI; cell density, epithelial cell density and epithelial fraction from histology). *fEES*: Fractional extracellular extravascular space, *fIC*:Fractional intracellular volume, *fVASC* Fractional vascular volume, *R* Radius, *VERDICT* Vascular, Extracellular, and Restricted Diffusion for Cytometry in Tumors, *WSI* Whole-slide imaging.

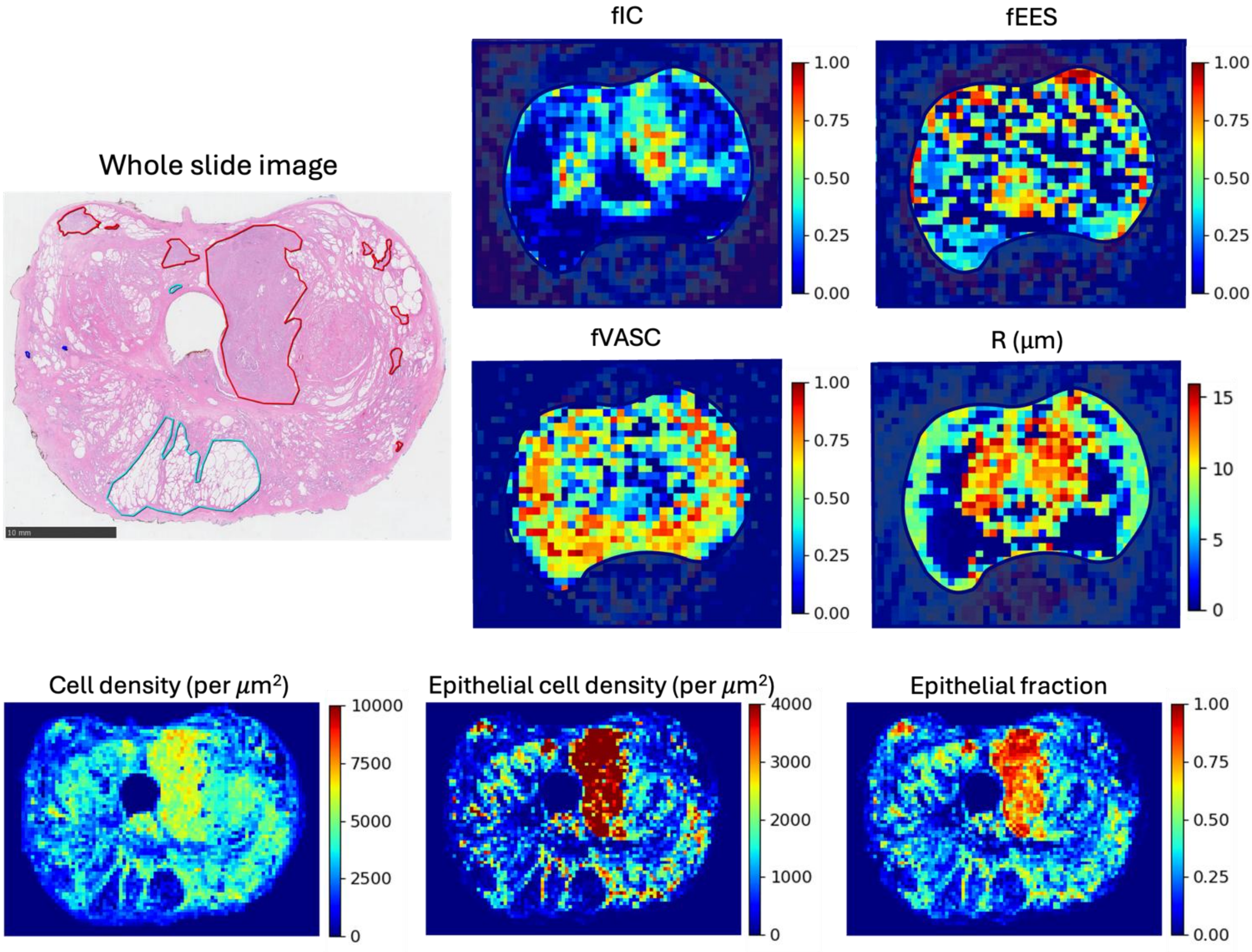

Fig. S2. Representative example: a 62-year-old man with prostate-specific antigen levels of 7.75 ng/mL at referral, prostate volume of 37 mL and Likert score 3. WSI (left) and corresponding masked MR and histologically derived maps (fIC, fEES, fVASC and R from VERDICT-MRI; cell density, epithelial cell density and epithelial fraction from histology). *fEES*: Fractional extracellular extravascular space, *fIC*:Fractional intracellular volume, *fVASC* Fractional vascular volume, *R* Radius, *VERDICT* Vascular, Extracellular, and Restricted Diffusion for Cytometry in Tumors, *WSI* Whole-slide imaging.
